# Competition between a noncoding exon and introns: *Gomafu* contains tandem UACUAAC repeats and associates with splicing factor-1

**DOI:** 10.1111/j.1365-2443.2011.01502.x

**Published:** 2011-05

**Authors:** Hitomi Tsuiji, Rei Yoshimoto, Yuko Hasegawa, Masaaki Furuno, Minoru Yoshida, Shinichi Nakagawa

**Affiliations:** 1 Nakagawa Initiative Research Unit, RIKEN Advanced Science InstituteHirosawa 2-1, Wako, Saitama 351-0198, Japan; 2 Chemical Geneticss Laboratory, RIKEN Advanced Science InstituteHirosawa 2-1, Wako, Saitama 351-0198, Japan; 3 RNA Biology Laboratory, RIKEN Advanced Science InstituteHirosawa 2-1, Wako, Saitama 351-0198, Japan; 4 Department of Chemistry and Biotechnology, Graduate School of Engineering, University of Tokyo7-3-1 Hongo, Bunkyo-ku, Tokyo 113-8656, Japan; 5 RIKEN Omics Science Center, RIKEN Yokohama InstituteKanagawa 230-0045, Japan; 6 PRESTO, Japan Science and Technology AgencyKawaguchi-shi, Saitama 332-0012, Japan

## Abstract

*Gomafu* (also referred to as *RNCR2/MIAT*) was originally identified as a noncoding RNA expressed in a particular set of neurons. Unlike protein-coding mRNAs, the Gomafu RNA escapes nuclear export and stably accumulates in the nucleus, making a unique nuclear compartment. Although recent studies have revealed the functional relevance of Gomafu in a series of physiological processes, the underlying molecular mechanism remains largely uncharacterized. In this report, we identified a chicken homologue of *Gomafu* using a comparative genomic approach to search for functionally important and conserved sequence motifs among evolutionarily distant species. Unexpectedly, we found that all Gomafu RNA examined shared a distinctive feature: tandem repeats of UACUAAC, a sequence that has been identified as a conserved intron branch point in the yeast *Saccharomyces cerevisiae*. The tandem UACUAAC Gomafu RNA repeats bind to the SF1 splicing factor with a higher affinity than the divergent branch point sequence in mammals, which affects the kinetics of the splicing reaction *in vitro*. We propose that the Gomafu RNA regulates splicing efficiency by changing the local concentration of splicing factors within the nucleus.

## Introduction

Growing evidence has demonstrated the functional importance of long non-protein-coding RNAs (lncRNAs), which constitute a significant fraction of the transcriptional output from the mammalian genome (reviewed in Prasanth & Spector 2007; Mercer *et al.* 2009). The best-characterized example among these is *Xist*, which regulates epigenetic dosage compensation by recruiting chromatin-modifying complexes to one of the two X chromosomes in female mammals (reviewed in Payer & Lee 2008). Other lncRNAs that regulate chromatin modification include *Airn* (Nagano *et al.* 2008), *HOTAIR* (Gupta *et al.* 2010), *Kcnq1ot1* (Pandey *et al.* 2008) and *p15AS* (Yu *et al.* 2008). Notably, epigenetic regulation of gene expression has been proposed to be one of the major functions of lncRNAs (reviewed in Nagano & Fraser 2009). Interestingly, recent high-throughput sequence analyses identified numerous promoter-associated transcripts in higher eukaryotes (Kapranov *et al.* 2007; Core *et al.* 2008; Preker *et al.* 2008; Seila *et al.* 2008) and yeast (Wyers *et al.* 2005; Davis & Ares 2006). These lncRNAs are expected to play an active role in the transcriptional control of neighboring genes (reviewed in Seila *et al.* 2009), although the physiological relevance of their expression remains to be experimentally validated.

Nuclei of higher eukaryotes are highly organized and can be divided into several nuclear compartments containing distinct sets of proteins that are essential for particular nuclear processes (reviewed in Spector 2001). For example, ribosome biogenesis occurs in the nucleolus (Boisvert *et al.* 2007); nuclear speckles contain a series of SR proteins and other splicing factors (reviewed in Lamond & Spector 2003); A-to-I edited mRNAs are retained in the paraspeckle, a recently identified nuclear compartment (reviewed in Bond & Fox 2009), and the Cajal bodies contain proteins required for snRNP maturation (reviewed in Gall 2003). Aside from the aforementioned lncRNA-mediated regulation of gene transcription, products of two abundant lncRNAs, which are denoted nuclear-enriched abundant transcript 1 [*NEAT1,* also referred to as *MENε/β* (Guru *et al.* 1997) or *VINC* (Saha *et al.* 2006)] and *NEAT2* [also referred to as *Malat1* (Ji *et al.* 2003)], have been shown to associate with particular nuclear compartments: the paraspeckles and nuclear speckles, respectively (Hutchinson *et al.* 2007). Importantly, depletion of the NEAT1/MENε/β RNA leads to the disintegration of paraspeckles and subsequent re-distribution of paraspeckle components, such as p54^nrb^, PSF1 and PSP1 (Clemson *et al.* 2009; Sasaki *et al.* 2009; Sunwoo *et al.* 2009). In addition, knockdown of Malat1/NEAT2 RNA causes de-localization of a certain group of SR proteins within the nuclear speckles (Tripathi *et al.* 2010). Therefore, one of the principal functions of lncRNAs might be to provide an architectural scaffold that is essential for the integrity of particular nuclear compartments, especially when considering the functions of the abundant lnRNAs in the nucleus.

*Gomafu/RNCR2* was originally identified as a noncoding RNA expressed in a specific set of neurons in the mouse retina (Blackshaw *et al.* 2004; Sone *et al.* 2007). *Gomafu* is widely and abundantly expressed in the nervous system throughout development, and its expression continues into adulthood (Sone *et al.* 2007). The Gomafu RNA escapes nuclear export, even though it has mRNA-like characteristics (i.e., polyadenylation and splicing) and accumulates within the nucleus, where it forms a novel structure that does not coincide with known nuclear compartment markers (Sone *et al.* 2007). Interestingly, single nucleotide polymorphisms in the human homologue of *Gomafu* are associated with an increased risk of myocardial infarction, and thus the gene has been named myocardial infarction associated transcript (*MIAT*) (Ishii *et al.* 2006). Recently, *Gomafu/RNCR2* has also been shown to control the differentiation of retinal cells (Rapicavoli *et al.* 2010) and the pluripotency of embryonic stem cells (Sheik Mohamed *et al.* 2010), although the underlying molecular mechanism remains completely unknown.

To obtain insight into the molecular function of *Gomafu*, we tried to identify functionally important, conserved sequence motifs in *Gomafu/RNCR2/MIAT*. We identified an evolutionarily distant chicken homologue of *Gomafu* using a comparative genomic approach. We found that all *Gomafu* genes from three different species contained a tandem repeat of TACTAAC, which is the essential and conserved intron branch point sequence in the budding yeast *Saccharomyces cerevisiae*. The UACUAAC repeat of the Gomafu RNA bound to splicing factor SF1 with a higher affinity than the mammalian branch point consensus sequences and inhibited the splicing reaction of a model substrate *in vitro*. We propose that the family of *Gomafu* lncRNAs constitute a novel nuclear domain that competes with sub-optimal intron branch point sequences for binding to the SF1 splicing factor.

## Results

### Identification of chicken *Gomafu* (*cGomafu*) using a comparative genomic approach

**Figure 1:**
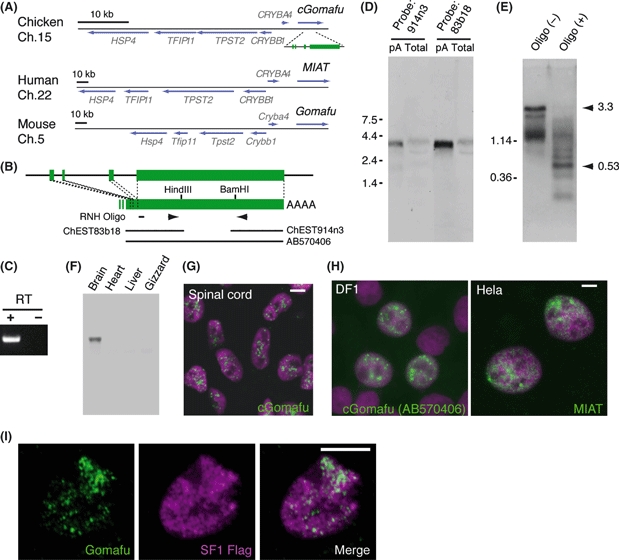
Identification of chicken *Gomafu* (c*Gomafu*) using a comparative genomic approach. (A) Schematic representation of the syntenic region of *CrybA4* in the chicken, human and mouse genomes. *Gomafu* is located 3′ to *CrybA4* in all three vertebrate species. (B) Genomic organization of c*Gomafu*. The positions of EST clones and oligonucleotides used for the RNase H treatment are indicated with bars. Arrowheads indicate the positions of primers that were used to amplify the overlapping middle fragment of c*Gomafu*. (C) Results of RT-PCR of the middle fragment of c*Gomafu*. (D) Northern blot analysis of *cGomafu* in 3 μg of poly-A (+) (pA) and 10 μg of total RNA (total) derived from E5 embryonic brains. Both of the probes prepared from two independent EST clones detected a single band of the same size. Note the split bands in the total RNA samples because of the ribosomal RNA. (E) RNase H northern blot analysis of Gomafu RNA digested with RNase H and the oligonucleotide shown in ‘B’. The RNase H digestion produced a major band of 0.53 kb. ChEST83b18 and its RNase H-digested 5′ fragment were 1.14 and 0.36 kb, respectively. (F) Multiple-tissue northern blot analysis of the *cGomafu*. *cGomafu* was specifically expressed in the brain. (G) Subcellular localization of the cGomafu RNA (green) in the spinal cord of E5 chicken embryos, as revealed by fluorescent *in situ* hybridization. (H) Subcellular localization of the cGomafu RNA and MIAT RNA exogenously expressed in the chicken cultured cell line DF1 and HeLa cells, respectively. Note that both of the overexpressed RNA transcripts (green) accumulated in the nucleus. Cellular nuclei were counter-stained with DAPI (magenta) in ‘G’ and ‘H’. (I) Simultaneous detection of the Gomafu RNA (green) and FLAG-SF1 (magenta). Most of the SF1 signals did not overlap with the Gomafu RNA signals. Scale bars, 10 μm.

To identify a *Gomafu/RNCR2/MIAT* homologue, we initially performed a BLAST search (http://blast.ncbi.nlm.nih.gov/Blast.cgi) against a nonredundant nucleotide database using the *Gomafu, RNCR2* and *MIAT* sequences as queries, which yielded no hits. We subsequently noticed that *Gomafu* and *MIAT* are positioned in syntenic regions of mouse chromosome 5 and human chromosome 22, respectively: 3′ to *Crystallin beta A4* (*CrybA4*) (Fig. 1A). We thus speculated the syntenic region is transcribed into a noncoding RNA. Using Map Viewer (http://www.ncbi.nlm.nih.gov/mapview/), we found that a number of EST clones were mapped to the genomic region 3′ to *CrybA4* of *Gallus gallus* (domestic chicken) (Fig. 1B). Northern blot analysis using probes prepared from two independent EST clones in that region, ChEST83b18 and ChEST914n3, revealed a single 3.3-kb band (Fig. 1D), suggesting that they were derived from the same gene product. This observation was further supported by the results obtained from RT-PCR using primers against the 3′ end of ChEST83b18 and 5′ end of ChEST914n3 (Fig. 1C). To determine whether ChEST83b18 covered the 5′ end of the transcript, we used northern blot analysis of a shorter RNA fragment, which was digested 0.36 kb downstream of the 5′ end of the EST clone (Fig. 1B, E). The major band at 0.53 kb was observed after digestion with RNase H, suggesting that ChEST83b18 lacked 0.17 kb of sequence from the 5′ end. To obtain the 5′ end of the transcript, we performed 5′ RACE analysis; however, repeated trials failed to reveal the upstream fragments, probably because of secondary structures or the GC-rich nature of the sequence. We then performed 3′ RACE analysis and found that the obtained clones contained the same 3′ end sequences and nongenomic poly-A sequences as the EST clone ChEST914n3. The transcripts were enriched in the poly-A (+) fractions (Fig. 1D), suggesting that the transcript was polyadenylated, although the genomic sequence did not appear to contain a common polyadenylation signal (AATAAA or ATTAAA). This gene was specifically expressed in the brain but not in other tissues, including the heart, liver and gizzard (Fig. 1F). We further investigated the subcellular localization of the transcript by fluorescent *in situ* hybridization (FISH) and found that the transcript was diffusely localized in the nuclei of spinal neurons (Fig. 1G), yielding a spotted pattern similar to that observed for the mouse Gomafu RNA (Sone *et al.* 2007). We designated this gene chicken *Gomafu* (*cGomafu*), because of its characteristic subnuclear distribution (Gomafu means ‘spotted pattern’ in Japanese) and specific expression in the nervous system. We then introduced a fragment of the *cGomafu* cDNA (AB570406), which lacked the short 5′ fragment, into the DF1-cultured chicken cell line and found that the *cGomafu* fragment transcript was localized in the nucleus (Fig. 1H). The full-length transcript of *MIAT* was also localized in the nucleus when overexpressed in HeLa cells (Fig. 1H), suggesting that nuclear localization of the transcript is a common feature of *Gomafu* homologues in different vertebrate species.

### Gomafu contains multiple TACTAAC tandem repeats and binds to SF1

**Figure 2:**
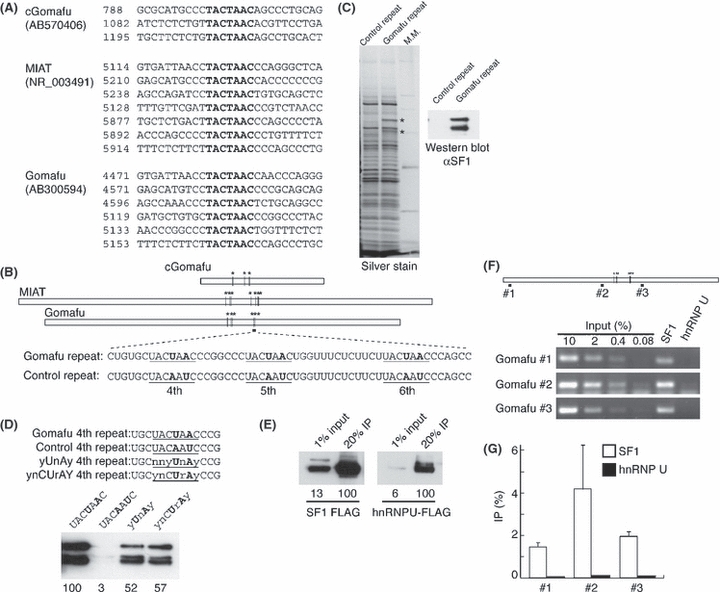
The Gomafu RNA contains tandem repeats of UACUAAC and binds to SF1. (A) Conserved TACTAAC repeats in *Gomafu* from different species. The numbers indicate the position in each cDNA clone. (B) Schematic representation of the TACTAAC repeats in *Gomafu* homologues and the sequences of the *Gomafu* repeats used for affinity purification experiments. Bold letters indicate the critical residues for branch point recognition that were mutated in the control. (C) Affinity purification of proteins associated with the Gomafu repeat RNA. SF1 was specifically precipitated by the Gomafu UACUAAC repeat. (D) Affinity purification of SF1 with various branch point sequences. Note that UACUAAC shows the highest affinity binding to SF1. The values below indicate the quantification of the western blot signals. (E-G) Specific interaction between SF1 and the Gomafu RNA. (E) Confirmation of the immunoprecipitation efficiency. FLAG-tagged SF1 or control hnRNP U was overexpressed in Neuro2A cells and immunoprecipitated by the anti-FLAG antibody. The values below indicate the quantification of the western blot signals. (F) Semi-quantitative RT-PCR analysis showing the interaction of the Gomafu RNA with SF1. (G) Quantification of the data shown in ‘E’.

To identify sequence motifs conserved among *Gomafu*, *MIAT* and *cGomafu*, we utilized the MEME algorithm (http://meme.sdsc.edu/meme/intro.html) and found that all three genes shared characteristic feature of having multiple TACTAAC repeats in tandem (Fig. 2A, B), but the overall sequence similarity was quite low. A text search against the Refseq database revealed that several well-characterized transcribed genes (those starting with NM_ or NR_) contained multiple TACTAAC sequences; however, the feature was not conserved among different species, except for *Gomafu* (Table 1). The UACUAAC, RNA sequence of TACTAAC, is a strictly conserved branch point consensus sequence and is essential for intron removal in the budding yeast *Saccharomyces cerevisiae* (Langford *et al.* 1984) (the branch point adenosine is underlined). We therefore considered that the tandem UACUAAC repeat in the Gomafu RNA might interact with Splicing factor 1 (SF1) (Kramer 1992), a vertebrate homologue of yeast branch point binding protein (BBP) that binds strongly to the UACUAAC sequence (Berglund *et al.* 1997). To test this idea, we synthesized biotinylated RNA oligos containing tandem repeats of the Gomafu RNA (5123–5175 of AB300594) and performed affinity purification using a nuclear extract from Neuro2A cells. As a control, we used the synthetic oligonucleotides with mutations in the two essential nucleotide sequences required for BBP/SF1 binding (UACAAUC; the mutations are underlined) (Berglund *et al.* 1997). Two specific proteins of 80 and 67 kD bound specifically to the Gomafu RNA fragment (Fig. 2C), and subsequent mass spectroscopy and western blot analysis confirmed that these bands represented SF1 (Fig. 2C).

**Table 1:** Refseq genes containing three or more TACTAAC repeats in vertebrates

Species	Repeat no.	Accession no.	Length (bp)	Gene
Human	7	NR_003491	10142	MIAT
	3	NM_032217	9390	ANKRD17
	3	NM_178123	10448	SESTD1
	3	NM_020122	7555	KCMF1
	3	NM_006197	8788	PCM1
Chicken	3	AB570406	3045	cGomafu
Mouse	8	NM_001112798	18563	Slc8a1
	8	NM_001131020	2600	Gfap*
	6	AB300594	8701	Gomafu/Miat/Rncr2
	4	NM_023662	8398	Pcm1
	4	NR_001461	83437	Kcnq1ot1
	3	NM_010252	4771	Gabrg1
	3	NM_011857	10978	Odz3
	3	NR_003549	33831	3110048L19Rik†
	3	NM_011652	101674	Ttn

* Rare transcript containing intron sequences.

† Zinc finger pseudogene.

The branch point sequence is highly diverged in higher eukaryotes; the consensus sequence is ynCUrAy in mammalian species (reviewed in Burge *et al.* 1999) and yUnAy in humans (n=A, U, C, G; y=C or U; r=A or G) (Gao *et al.* 2008). A recombinant fragment of SF1 consistently binds to UACUAAC and the mutated sequences in a similar manner as long as the two critical residues are conserved (Berglund *et al.* 1997). However, UACUAAC from the forth Gomafu repeat showed decreased binding affinity with SF1 when the sequence was mutated to ynCUrAy or yUnAy (Fig. 2D), even though the critical adenosine and uridine residues were present. This observation agrees with a previous report that the artificial UACUAAC sequence serves as an optimal branch point sequence in mammals (Zhuang *et al.* 1989) and suggested that the Gomafu RNA provides a higher affinity binding site for SF1 compared to the intron branch point sequences of endogenous pre-mRNAs.

To further confirm that SF1 interacts with Gomafu RNA *in vivo*, we performed immunoprecipitation RT-PCR experiments using Neuro2A cells that express cDNA of *Gomafu* without introns and SF1 tagged with a FLAG epitope (Fig. 2E–G). Because Gomafu RNA was present in an insoluble fraction called the nuclear matrix (Sone *et al.* 2007), we solubilized the RNA-protein complex using mild sonication in denaturing conditions containing 1% SDS after the cross-link by UV irradiation, a method that has been used to detect the interaction between the Xist RNA and its interacting protein hnRNP U (Hasegawa *et al.* 2010). Under these conditions, the Gomafu RNA was specifically immunoprecipitated (Fig. 2F, G), suggesting that the Gomafu RNA binds directly to SF1 *in vivo*. We also examined whether the Gomafu RNA and FLAG-tagged SF1 colocalized in the nucleus by simultaneous detection of FISH and immunofluorescence signals. SF1-FLAG was broadly distributed in the nucleus, whereas Gomafu RNA was observed as discrete dots (Fig. 1I), suggesting that Gomafu RNA interacts with a small fraction SF1.

### TACTAAC repeat in *Gomafu* is not necessary for nuclear localization

**Figure 3:**
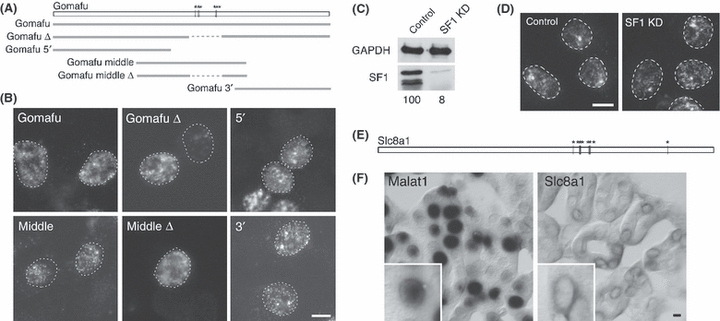
Nuclear localization signals are redundantly distributed in the Gomafu RNA. (A) Schematic drawing of the Gomafu RNA fragments introduced into the Neuro2A cells. Asterisks show the positions of the seven UACUAAC repeats in the Gomafu RNA. (B) Subcellular localization of the Gomafu RNA fragments revealed by FISH. All of the Gomafu RNA fragments were localized in the nucleus, regardless of the presence of the UACUAAC repeat. (C) Western blot analysis of siRNA-mediated depletion of SF1 in the Neuro2A. The values below indicate the quantification of the western blot signals. (D) Distribution of Gomafu RNA in the SF1-depleted cells. Note that the nuclear localization was not affected by the SF1 knockdown. (E) Schematic drawing of the structure of *Slc8a1*. Asterisks show the positions of the eight UACUAAC sequences. (F) Localization of *Malat1* and *Slc8a1* transcripts in adult kidney cortex. The *in situ* hybridization signals were detected using the NBT/BCIP development method. Note that Slc8a1 transcripts are predominantly distributed in the cytoplasm surrounding the nucleus. Insets show higher magnification images. Scale bars, 10 μm.

Because the tandem TACTAAC repeats were the only conserved feature found in the *Gomafu* homologues in different vertebrate species, we speculated that this sequence might regulate nuclear localization. We therefore stably transfected fragments of *Gomafu* that did or did not contain the TACTAAC repeats into Neuro2A cells (Fig. 3A, B). All five of the fragment of Gomafu RNA as well as the full-length Gomafu RNA were localized to the nucleus when overexpressed in Neuro2A cells (Fig. 3B), suggesting that the nuclear localization elements are widely distributed throughout the Gomafu RNA and that the repeats are not necessary for the nuclear localization of 5′ and 3′ fragments of Gomafu RNA. We further examined the effect of SF1 knockdown on the Gomafu RNA (Fig. 3C, D) to determine whether SF1 regulates nuclear retention or stability of the Gomafu RNA. The siRNA efficiently depleted SF1 (Fig. 3C); however, stability or subcellular localization of the Gomafu RNA was not significantly influenced (Fig. 3D), suggesting that SF1 might act downstream of the Gomafu RNA rather than regulating its stability or localization. We also examined the expression of *Slc8a1*, which contains eight tandem TACTAAC repeats (Table 1, Fig. 3E). Unlike nuclear-localizing Malat1 RNA, transcripts of *Slc8a1* were predominantly localized to the cytoplasm (Fig. 3F). These signals were not detected with sense probes for *Slc8a1* (data not shown). Thus, the presence of multiple UACUAAC sequences was not sufficient for the nuclear retention of mRNA.

### TACTAAC repeat in *Gomafu* delays splicing kinetics *in vitro*

**Figure 4:**
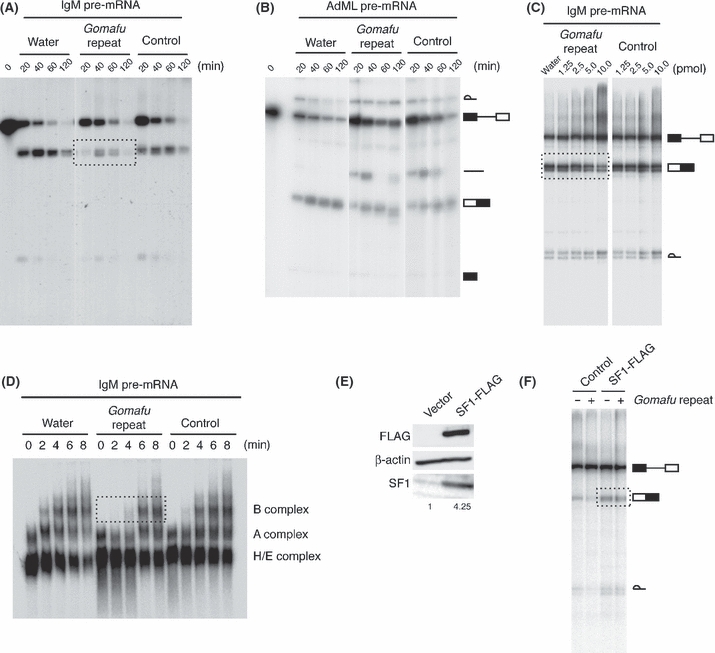
TACTAAC repeats in Gomafu delay splicing kinetics *in vitro*. (A, B) *In vitro* competition experiments using the *Gomafu* repeat oligonucleotides. A HeLa cell nuclear extract was pre-incubated on ice, with either water or the oligonucleotides (5 pmol/reaction) used in Fig. 2C. After the addition of the IgM pre-mRNA (A) or AdML pre-mRNA (B), the mixture was incubated at 30 °C for the indicated time. The bands for the RNA products are shown schematically at the right. Note that a marked decrease in the spliced product was observed with the IgM pre-mRNA (dashed box) but not with AdML pre-mRNA. (C) Dose-dependent inhibition of IgM pre-mRNA splicing by the *Gomafu* repeat oligonucleotides. The *in vitro* splicing reaction was performed in the presence of water or indicated amount of oligonucleotides at 30 °C for 60 min. Gomafu repeat but not control oligonucleotides inhibited the formation of spliced product in a dose-dependent manner (dashed box). Note that lariat intron is stabilized in the presence of higher amount of oligonucleotides, probably due to an inhibition of endogenous nucleases. (D) Analysis of splicing complex formation. Splicing complexes from the same reaction conditions as in ‘A’ were separated on a native 2% agarose gel. Formation of complex B was significantly retarded in the presence of the *Gomafu* repeat oligonucleotides. (E) Expression of SF1 in HEK293T cells transfected with control or SF1-expressing vector. Total protein from an equivalent number of cells was separated by 8% SDS–PAGE and detected on the Western blot using anti-FLAG, anti-β-actin and anti-SF1 antibody. The numbers below indicate the relative amount of SF1. (F) Neutralization of inhibitory effect of Gomafu repeat oligonucleotides by an excess amount of SF1. HEK293T cell nuclear extract expressing either empty vector or SF1-FLAG protein was pre-incubated on ice with water or the indicated oligonucleotides (5 pmol/reaction). After the addition of the IgM pre-mRNA, the mixture was incubated at 30 °C for 60 min. Exogenous SF1 protein rescues the splicing efficiency of IgM pre-mRNA (dashed box).

Considering the interaction between the Gomafu RNA and SF1, we hypothesized that the Gomafu RNA might regulate splicing by competing locally with the branch point sequences of pre-mRNAs for the splicing factor SF1. To confirm this hypothesis, we examined the effect of UACUAAC tandem repeats in the Gomafu RNA on the splicing reaction and spliceosome formation *in vitro*. We first used a model pre-mRNA substrate derived from mouse IgM (Watakabe *et al.* 1993), which possesses a predictably weak branch point with a degenerate sequence (Guth & Valcarcel 2000). As expected, the addition of the *Gomafu* repeat oligonucleotides when compared with the control oligonucleotides markedly delayed production of the spliced product (Fig. 4A). We then used another pre-mRNA substrate with strong intron consensus sequences derived from adenovirus (Zapp & Berget 1989). In this case, both the control and *Gomafu* repeat oligonucleotides inhibited the splicing reaction to some extent; however, no differences were found between the two conditions (Fig. 4B). These results were consistent with previous reports showing that BBP and SF1 are not essential for the splicing reaction itself but are required for optimal removal of introns with sub-optimal consensus sequences (Guth & Valcarcel 2000; Rutz & Seraphin 2000; Tanackovic & Kramer 2005). We then examined whether the *Gomafu* repeat oligonucleotides inhibit IgM pre-mRNA splicing in a dose-dependent manner. In this experiment, the splicing reaction was performed in an increasing amount of oligonucleotides for a fixed time (60 min). As expected, control oligonucleotides did not inhibit pre-mRNA splicing within a range of 1.25–10 pmol/reaction. On the other hand, inhibitory effect was recognizable with *Gomafu* repeat oligonucleotitdes as little as 2.5 pmol/reaction, which became clearer with an increased amount of the oligonucleotides (Fig. 4C). We also examined the effect of the *Gomafu* repeat oligonucleotides on the formation of a spliceosome complex using native gels. Although we did not observe obvious delay in the formation of H/E or A complex, the formation of B complex was markedly delayed in the presence of the *Gomafu* repeat oligonucleotides (Fig. 4D). Finally, we tested whether these inhibitory effects could be neutralized by an excess amount of SF1 using nuclear extracts prepared from HEK293T cells overexpressing SF1 (Fig. 4E, F). The overexpression resulted in approximately 4 times more SF1 compared with the control cells (Fig. 4E). As expected, the inhibitory effect of the Gomafu repeat oligonucleotides was rescued when using nuclear extracts prepared from SF1-overexpressing cells (Fig. 4F). Taken together, these results suggested that Gomafu RNAs potentially affect kinetics of splicing reaction by competing with endogenous introns for the branch point binding protein SF1.

## Discussion

We demonstrated here that *Gomafu* is an lncRNA that is conserved among higher vertebrates, including human, mouse and chicken, in terms of its characteristic nuclear localization as well as its specific expression pattern in the nervous system. Because no syntenic chromosomal region has been identified in other vertebrate species, we were not able to identify more *Gomafu* homologues using the comparative genomic approach described here. While preparing this manuscript, Blaskshaw and colleagues reported that *RNCR2* contains tandem repeats of ACTAACY (Rapicavoli *et al.* 2010), which mostly overlapped with the TACTAAC repeat of *Gomafu* identified here. Based on the observation of multiple ACTAACY sequences, the authors proposed that the clawed frog *Xenopus tropicalis* possesses a homologue of *RNCR2* (Rapicavoli *et al.* 2010). It would be intriguing to study whether the frog gene is specifically expressed in the nervous system and whether the transcripts are localized in the nucleus; these are the two criteria used to define the *Gomafu* lncRNA family.

Using the MEME algorithm, we determined that all three *Gomafu* homologues share a unique characteristic: a tandem repeat of TACTAAC, which has long been recognized as a consensus intron branch point sequence in the budding yeast *Saccharomyces cerevisiae*. Considering that the branch point sequence is highly divergent in higher eukaryotes, it is particularly interesting that the ‘intronic’ sequence of the single-cell budding yeast is conserved in the ‘exon’ of evolutionarily distant, multicellular organisms. Higher affinity binding of the UACUAAC sequence to SF1 may explain this apparent discrepancy. The budding yeast uses this sequence to minimize the size of introns with strong branch point sequences, resulting in the strict definition of intron positions. On the other hand, higher vertebrate species, such as mammals and birds, use tandem repeats of this sequence to regulate splicing events by inserting repeats into the exon of the *Gomafu* lncRNA, which stably accumulates in the nucleus and facilitates local regulation of the SF1 concentration. It should be noted that SF1 is not essential for the splicing reaction *per se*, but it modulates the efficiency of splicing kinetics, especially if the intron consensus sequence is sub-optimal (Guth & Valcarcel 2000; Rutz & Seraphin 2000; Tanackovic & Kramer 2005). The splicing regulation mediated by the Gomafu RNA and SF1 may increase the complexity of alternative splicing events observed in higher eukaryotes, which are thought to be the basis for the functional diversity in metazoan organisms (reviewed in Blencowe 2006). It should also be stressed that the distribution of the Gomafu RNA and SF1 did not coincide perfectly. Therefore, the Gomafu RNA is expected to affect a few splicing events, if any, that are regulated by SF1. Regardless, the regulation of splicing might be an essential target of nuclear-enriched, stable lncRNAs that appeared recently in the history of the evolution.

## Experimental procedures

All the primer information is provided in Data S1 (Supporting information).

### cDNA cloning and vector construction

The middle region of *cGomafu* was amplified with the primer #1 and #2 and cDNAs derived from E5 embryonic chicken brain as a template. The resultant fragment was ligated to ChEST83B18 and ChEST914n3 using the HindIII and BamHI sites to yield the longest cDNA clone of *cGomafu*. The partial cDNA fragment lacking the short 5′ sequence was subcloned into pT2K-CAGGS (Y) (Sato *et al.* 2007) to generate the expression vector for *cGomafu*. To confirm the 3′ end sequence of *cGomafu*, 3′ RACE was performed using the SMART RACE kit (Clontech), according to the manufacturer's instructions and a gene-specific primer #3. To obtain the full-length cDNA clone of MIAT, the 5′ region of this gene was amplified with the primers #4 and #5 and the BAC clone RP11262F9 as a template. The fragment was ligated to a 2.3-kb *MIAT* fragment that covered the exon–exon junction of *MIAT* (Ishii *et al.* 2006), using a SacII site. The middle region of *MIAT* was then amplified with the primers #6 and #7 and the BAC clone RP11262F9 as a template. This region was then ligated to the 5′ fragment using the NcoI site. The resultant fragment was then ligated to AK127256, which contained the 3′ end of *MIAT*, using the SphI site. The full-length cDNA was then subcloned into pT2K-CAGGS (Y) to generate the expression vector for MIAT. AK028326 and AK053540 were subcloned into pT2K-CAGGS (Y) to generate the plasmids for the expression of the 5′ and 3′ fragments of *Gomafu*, respectively. For the middle fragment expression vector, the fragment was amplified with the primers #8 and #9 and then subcloned into pT2K-CAGGS (Y). For the middle fragment without the TACTAAC tandem repeat, a region corresponding to 4581–5169 of AB300594 was deleted by DpnI-mediated site-directed mutagenesis (Weiner *et al.* 1994). To generate the FLAG-tagged SF1, the full-length open reading frame lacking the stop codon was amplified by PCR and then subcloned into pCAGGS-FLAG (Hasegawa *et al.* 2010).

### Northern blot analysis and RNase H treatment

Northern blot analysis was performed according to a standard protocol using DIG-labeled RNA probes. Total RNA was isolated from E18 chicken embryos using Trizol (Invitrogen), and poly-A (+) RNA was purified using the Oligotex dT-30 (super) mRNA isolation kit (Takara, Japan); 10 μg of total RNA or 3 μg of poly-A (+) RNA was used for the Northern blot analysis. For the RNase H treatment, 3 μg of poly-A (+) RNA was mixed with 25 pmol of oligonucleotide #10 and heated at 65 °C for 5 min. Samples were then treated with RNase H (Toyobo, Japan) for 30 min at 37 °C and subjected to northern blot analysis using an 1.5% agarose gel. DIG-labeled probes were prepared from the EST clones ChEST83b18 and ChEST914n3.

### *In situ* hybridization

DF1 and HeLa cells were transfected with the *cGomafu* and *MIAT* expression plasmids, respectively. A Tol2 transposon-mediated gene transfer method was employed, which facilitates convenient introduction of exogenous genes into the host genome of cultured cells (Sato *et al.* 2007). The cultured cells were transfected with a mixture of the pT2K expression vectors and pCAGGS-T2TP using Fugene (Roche) and then cultured for 10 days, at the timing where the introduced genes were stably integrated into the genome. Fluorescent *in situ* hybridization was performed as previously described (Sone *et al.* 2007). Probes for *cGomafu* and *Slc8a1* were prepared from the EST clones ChEST83b18 and AK048160, respectively. To detect *MIAT*, a cDNA fragment without the repeat sequences was amplified with the primers #11 and #12 and AK127256 as a template and then subcloned into pCRII (Invitrogen). For simultaneous detection of SF1 and Gomafu RNA, FLAG-tagged SF1 was stably introduced into Neuro2A cells using the Tol2 system. The following antibodies were used: mouse monoclonal anti-DIG antibody (Roche), mouse monoclonal anti-FLAG antibody (Sigma), Cy3-conjugated anti-mouse antibody (Chemicon), rabbit polyclonal anti-FITC antibody (Invitrogen), Alexa Fluor 488-conjugated anti-rabbit antibody and alkaline phosphatase-conjugated sheep anti-DIG antibody (Roche). The images were obtained using an epifluorescent microscope (BX51; Olympus) equipped with a CCD camera (DP70).

### Affinity purification of SF1 from cell lysates

The synthetic RNA probes (#13–#18) were purchased from GeneDesign (Japan). All probes were labeled with biotin at the 3′ end during synthesis. To prepare the probe-conjugated beads, 1.8 nmol of biotinylated RNA was incubated with 30 μL of streptavidin-agarose (Fluca) for 2.5 h at 4 °C in 300 μL of binding buffer (1 m NaCl, 50 mm Tris, pH 7.4, 5 mm EDTA and 0.1% Triton X-100). After washing with the same buffer, the beads were equilibrated with RIPA (50 mm Tris, pH 7.5, 150 mm NaCl, 0.25% sodium deoxycholate and 1% Triton X-100) containing 1.2 U/μL of RNase inhibitor (Toyobo). Neuro2A cells were grown to confluence on a 10-cm culture dish (Nunc) and suspended in 1 mL of RIPA. The cell suspensions were sonicated for 5 s at maximum power (UR-20P; Tomy Seiko Co., Ltd.) and centrifuged at 12 000 ***g*** for 20 min. The cell lysates were pre-cleared with 100 μL of streptavidin beads for 3 h at 4 °C and incubated with 30 μL of RNA-conjugated beads overnight at 4 °C. After five washes with RIPA, bound proteins were eluted with 40 μL of RNase A/T1 in 1:2 diluted RIPA for 30 min at 37 °C and then used for subsequent SDS–PAGE analysis. For Western blotting, rabbit polyclonal anti-SF1 (Sigma) and HRP-conjugated anti-mouse IgG (GE Healthcare) were used. Western blot signals were quantitated using VersaDoc (Biorad).

### Immunoprecipitation and RT-PCR

Immunoprecipitation and RT-PCR were performed as previously described (Hasegawa *et al.* 2010). Briefly, Neuro2a cells were transfected with the FLAG-tagged SF1 expression plasmid using Fugene (Roche). Forty-eight hours after transfection, cells were washed twice with Hepes-buffered saline (HBS; 10 mm Hepes at pH 7.4) and irradiated with 4 000 J/m^2^ UV in 1 mL of ice-cold HBS. The cells were collected in 1.5-mL microtubes and centrifuged; the cell pellets were re-suspended with 200 μL SDS buffer (50 mm Tris–HCl, pH 8.0, 1 mm EDTA, 150 mm NaCl, 1 mm DTT, 1% SDS and 1% Triton X-100). The cell lysates were then gently sonicated (UR-20P; Tomy Seiko Co., Ltd.) and diluted 10 times with a dilution buffer (50 mm Tris–HCl, pH 8.0, 1 mm EDTA, 150 mm NaCl, 1 mm DTT and 1% Triton X-100), which was supplemented with 1× protease inhibitor cocktail (Nacalai, Japan) and RNase inhibitor (Toyobo). After centrifugation, the soluble fraction was used for immunoprecipitation with 50 μL of anti-FLAG M2-Agarose (Sigma). The beads were washed once with a high-salt buffer (20 mm Tris–HCl, pH 8.0, 1 mm EDTA, 500 mm NaCl, 1 mm DTT, 0.1% SDS and 1% Triton X-100) and four times with a low-salt buffer (20 mm Tris–HCl, pH 8.0, 1 mm EDTA, 150 mm NaCl, 1 mm DTT, 0.1% SDS and 1% Triton X-100). After digestion with Proteinase K (PCR grade; Roche), the RNA was extracted with the Trizol Reagent (Invitrogen). RT-PCR was performed with ExTaq (Takara) using primers #19–#24.

### *In vitro* transcription and *in vitro* splicing assays

*In vitro* transcription was performed with either SP6 or T7 RNA polymerase. The *in vitro* splicing reaction was performed as described previously (Yoshimoto *et al.* 2009). Briefly, a typical 10-μL reaction mixture contained 3 μL of HeLa cell nuclear extract, 5 pmol of RNA oligo, 1 μL of 10 × SP and 2 μL of ^32^P-labeled transcript. In some experiments, nuclear extracts were prepared from HEK293T cells transfected with control or SF1-FLAG expressing vectors and were used for the *in vitro* splicing reaction. Note that the reaction mixtures were pre-incubated for 15 min on ice before the addition of the labeled transcript. Native gel analysis of the splicing complexes was performed according to the protocol described previously (Das & Reed 1999).The

## Supporting Information/Supplementary material

The following Supporting Information can be found in the online version of the article:

**Data S1** Primers used in this study.
